# The Recurrence of Venous Thromboembolism in Obstructive Sleep Apnea: A Narrative Review

**DOI:** 10.1155/carj/8848869

**Published:** 2025-07-10

**Authors:** Mohsen Gholinataj Jelodar, Besharat Rahimi, Samaneh Mirzaei

**Affiliations:** ^1^Department of Internal Medicine, School of Medicine, Shahid Sadoughi University of Medical Sciences and Health Services, Yazd, Iran; ^2^Clinical Research Development Center, Shahid Rahnemoon Hospital, School of Medicine, Shahid Sadoughi University of Medical Sciences and Health Services, Yazd, Iran; ^3^Advanced Thoracic Research Center, Tehran University of Medical Sciences, Tehran, Iran; ^4^Department of Health in Disaster and Emergencies, School of Public Health, Shahid Sadoughi University of Medical Sciences and Health Services, Yazd, Iran

**Keywords:** endothelial dysfunction, inflammation, obstructive sleep apnea, oxidative stress, recurrence, venous thromboembolism

## Abstract

Venous thromboembolism (VTE) is widespread and poses significant risks of illness and death, making it a vital public health issue. Obstructive sleep apnea (OSA), which is the most prevalent sleep disorder, is connected to an increased possibility of cardiovascular diseases and VTE. The length of VTE treatment hinges mainly on the frequency of its recurrence in patients. Our data about VTE and its recurrence in OSA patients are limited. In this review, we aim to investigate the risk of VTE recurrence in OSA patients and evaluate the role of continuous positive airway pressure (CPAP) therapy in mitigating this risk. A literature search gathered information about VTE pathogenesis and its potential recurrence mechanism in OSA. The recurrent episodes of partial or complete obstruction of the upper airway in OSA lead to intermittent lack of oxygen. Hypoxemia acts as a central cornerstone of VTE incidence in OSA patients, leads to activating all the vertices of Virchow's triad, and creates the appropriate condition for the developmental and even recurrence of VTE. Intermittent hypoxia causes an increase in the inflammatory state and coagulation activity, leading to oxidative stress and endothelial dysfunction. Furthermore, it results in heightened viscosity and venous stasis. The results of previous studies on VTE recurrence in OSA patients are conflicting. Even though the use of CPAP leads to diminished proinflammatory cytokines and oxidative stress indicators, there is currently insufficient clinical evidence to support that this therapy can prevent recurrent VTE in patients with OSA. Further investigation is necessary to gain a better comprehension of the probability and frequency of relapse of VTE in OSA patients, as the present research has generated inconclusive outcomes.

## 1. Introduction

Venous thromboembolism (VTE) is a medical condition that encompasses deep venous thrombosis (DVT) and pulmonary embolism (PE) [1, 2] and can cause significant morbidity in patients [3]. It is the third most common cause of death from vascular diseases [4], and up to 20% of patients die 1 year after diagnosis [5]. Approximately 500,000 cases of VTE-related deaths are estimated to occur annually in both the United States and Europe [4, 6]. The incidence rate differs across countries and is subject to variations based on age, gender, race, and clinical conditions [7]. About 2 in every 1000 people are affected by VTE annually [8]. Developed countries have a higher incidence rate than developing countries with lower income [9]. Among different racial groups, the incidence of VTE was highest in the Northern European population and lowest in Asians [10, 11]. In recent years, the incidence and mortality rate have decreased with the progress in diagnostic methods and the treatment of VTE patients [12]. VTE is a critical public health concern due to its morbidity, mortality, and financial burden [13].

Obstructive sleep apnea (OSA) is known as one of the most common sleep disorder [14, 15]. It is highly prevalent in the general population, and numerous cases are undiagnosed [16]. It has been reported that up to 30% of the US population is affected by this disorder [17], and according to studies, 2%–4% of the adult population in Western countries have symptomatic OSA [18]. There are about one billion people worldwide with OSA, and 425 million of them have moderate-to-severe symptoms [19]. It occurs two to three times more frequently in men than in women [20–23], and women's risk increases after menopause [24]. Episodes of partial or complete upper airway obstruction occur during sleep [25], leading to nocturnal hypoxemia, hypercapnia, and daytime sleepiness [25, 26]. OSA is a significant risk factor for cardio-cerebrovascular diseases, including hypertension (HTN), ischemic heart disease (IHD), heart failure [27], atrial fibrillation (AF), and stroke [28–30]. There is growing evidence that OSA is a risk factor for VTE [31–35]. Bahar et al. [36] conducted a study on the frequency of DVT in OSA patients, demonstrating a direct correlation between OSA severity and DVT risk. These findings support the hypothesis that OSA may independently contribute to thromboembolic events. In a study conducted by Kezban et al. [37] involving 30 PTE patients, 56.7% were found to have OSA, with 26.7% exhibiting moderate-to-severe cases (apnea-hypopnea index (AHI) > 15). Notably, among PTE patients without significant risk factors, OSA was the only independent risk factor identified (*p*=0.049). These findings highlight the necessity of considering OSA during risk assessment for PTE, particularly in patients lacking other traditional risk factors. It has also been seen that the prevalence of OSA is higher in patients with a history of previous VTE [38, 39]. A study reported that patients with PE had more snoring and a higher score on the Berlin Questionnaire [40]. Therefore, these two clinical conditions seem closely related and accompanied.

Obesity is recognized as the most important risk factor for developing OSA, which is seen in more than half of OSA cases [41]. Both neuromuscular and mechanical effects caused by tissue structural changes created in the neck region led to a greater tendency to obstruction and collapsibility in the upper airways [42–45]. Obesity, physical inactivity, and aging are shared risk factors for both VTE and OSA [35].

Historically, Virchow's triad has been described as the mechanism of pathogenesis in VTE, introduced by Rudolf Virchow in 1856. This triad includes a state of increased coagulability, venous stasis, and vascular endothelial cell damage [46, 47]. This triad is still the best justification for developing VTE in patients.

Despite previous research, our knowledge of the precise relationship between OSA and VTE recurrence remains limited and contradictory. Based on the abovementioned considerations, this narrative review aims to provide comprehensive information on the potential links between VTE and OSA. The narrative review primarily focuses on the pathogenesis of thrombosis in OSA patients and the reasons for their vulnerability to VTE based on the Virchow triad. Given the scarce information on the possibility of VTE recurrence in these patients, we will explore the potential for recurrence and its underlying factors.

## 2. Search Strategy

We conducted an electronic search to identify the appropriate articles based on the goal of this review. The databases, including Medline, PubMed, Web of Science, Scopus, and Google Scholar, covered the literature from inception to May 2024. The search was limited to articles published in English. The keywords used in our research were “DVT, thrombosis, venous thrombosis, deep venous thrombosis, VTE, venous thromboembolism, PE, pulmonary embolism, OSA, sleep apnea, obstructive sleep apnea, CPAP, NIV, continuous positive airway pressure, non-invasive ventilation, relapse, recurrence, recurrent thrombosis, and recurrent emboli.”

We included animal and human studies, clinical trials, cohort studies (both retro or prospective), case studies, reviews, and meta-analyses. Two authors reviewed the articles' titles and abstracts. When they could not reach an initial consensus, they reached the final decision through discussion or the opinion of the third author. Furthermore, the full text of the eligible articles was reviewed, and their bibliographies were analyzed to identify additional relevant studies.

## 3. The Role of Virchow's Triad in the VTE Formation in OSA Patients

### 3.1. Hypercoagulability State as a Result of Inflammation

There is evidence of the interaction of inflammation and thrombosis in the body. Indeed, many VTE risk factors act through inflammatory pathways that lead to thrombosis [48]. Lutsey et al. [49] evaluated the association of elevated C-reactive protein (CRP) with the incidence of VTE in 10,505 participants in the ARIC cohort study. The study concluded that the increased CRP level was an independent factor in the increased risk of VTE. For the first time, Engelmann and Massberg proposed immune thrombosis as a new term, which described the connection between inflammation and thrombosis [50]. According to the available findings, VTE is considered an immunothrombotic response [51]. Evidence of its increase has been seen in disorders with increased inflammation, such as the recent Coronavirus disease (COVID-19) pandemic [52]. Thrombosis is caused by the interaction of endothelial cells, platelets, and immune cells, which are regulated by different chemokines and cytokines [53]. It has been proposed that intermittent and chronic hypoxia during OSA disease is the main factor behind the increase in inflammatory activity among these patients [54].

Various episodes of hypoxemia in these patients lead to an increase in proinflammatory cytokine secretion [55–58]. Inflammatory cytokines are secreted from endothelial cells, platelets, monocytes, lymphocytes, natural killer (NK) cells, and antigen-presenting cells (APCs) [59, 60]. Secreted proinflammatory cytokines including interferon-gamma (INF-γ) [61, 62], interleukin (IL) 6 [63], IL-1 beta [64, 65], IL-17 [66], and transforming growth factor (TGF) beta [67] play a prominent role in the development of thrombosis.

**Figure 1:**
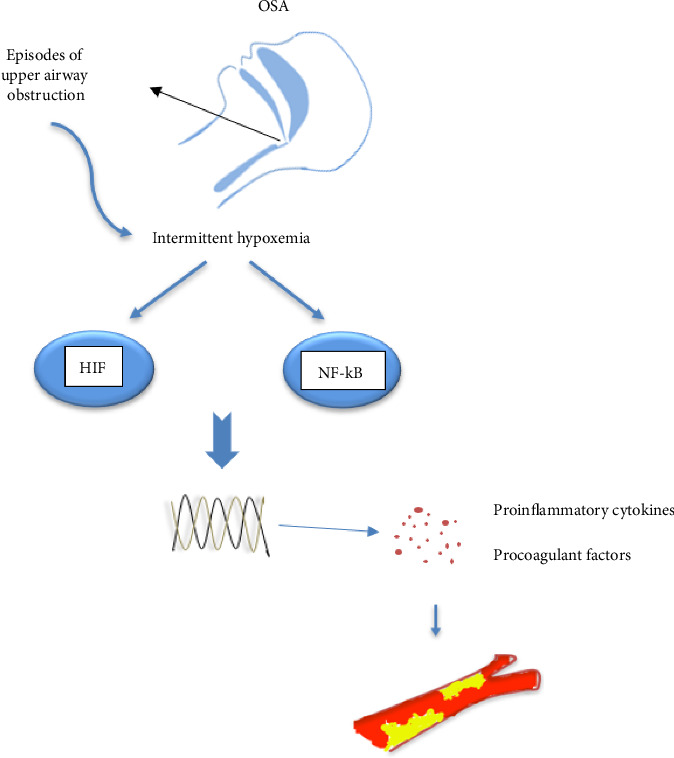
The link between inflammatory pathways and hypercoagulable states in OSA patients: Intermittent hypoxemia due to repetitive upper airway obstruction can ultimately activate the NF-κB and HIF family pathways. They induce genes that modulate the immune system and thrombosis. The secretion of various proinflammatory cytokines and procoagulant factors provides a favorable environment for developing intravascular thrombosis. OSA: obstructive sleep apnea, HIF: hypoxia-inducible factor, and NF-κB: nuclear factor kappa B.

Hypoxia has been shown to increase the activity of nuclear factor kappa B (NF-κB) and hypoxia-inducible factor-1 (HIF-1) pathways in patients with sleep apnea [68, 69] (Figure 1). In hypoxia, these two pathways interact [70, 71]. Increased NF-κB activity has been seen in immune and venous endothelial cells of OSA patients [72, 73]. Increased activity in the NF-KB inflammatory pathway leads to an increase in the secretion of ILs 1, 6, and 8, tumor necrosis factor (TNF) alpha, intracellular adhesion molecule-1 (ICAM-1), and vascular cell adhesion molecule (VCAM), ultimately resulting in a higher incidence of thrombosis [74–79]. Although the HIF-1 alpha pathway is known for regulating hypoxia adaptation genes [80], research has also shown its importance in inflammatory pathways [81, 82]. The HIF family includes three members: HIF1, HIF2, and HIF3. HIF 1 is the most important member of this family and is expressed in most tissues [83, 84]. HIFs are heterodimeric transcription factors comprising alpha and beta subunits. The alpha subunit expression varies based on the tissue oxygenation level and increases in hypoxemic conditions, with nuclear levels rising. By connecting to hypoxia-response elements (HRE) in target genes, they activate the transcription of many genes [85]. Similar to chronic hypoxia, long-term intermittent hypoxia has been shown to increase HIF-1 activity [86]. However, the signaling pathway for HIF1 activation in chronic hypoxia differs from intermittent hypoxia [87]. Its activation leads to the upregulation of over 100 genes, which, besides their role in various processes, are also effective in inflammation and immunity [88–90]. Recently, it has been shown that in patients with severe OSA during intermittent hypoxia, increased HIF-1α expression leads to increased nucleotide-binding oligomerization domain-like receptor 3 (NLRP3) inflammasome. The activity of the NLRP3 inflammasome complex plays a key role in the immune response, increasing the secretion of IL1 beta, IL18, and tissue factor [91] and enhancing the probability of VTE [92]. Activation of NLRP3 and IL-1 beta was involved in hypoxia-induced thrombosis in an animal study by Gupta et al. The pharmacological inhibition of this hypoxia-inflammatory pathway resulted in the transcription of NLRP3, a decrease in IL1 beta, and a significant antithrombotic effect [93].

Inflammation leads to the synthesis and secretion of tissue factors from endothelial cells and monocytes, which triggers the coagulation cascade in these patients [94]. Hypoxia leads to increased platelet function and disruption of fibrinolysis [31, 64]. Impaired fibrinolysis may be caused by increased plasminogen activator inhibitor-1 (PAI-1) levels in OSA patients [95–97]. PAI-1 has a significant role in regulating fibrinolytic activity and preventing clot dissolution [98]. Inflammatory pathways also increase PAI-1 levels. Studies show that TNF alpha and IL-6 have caused a rise in the expression of PAI-1 [99–103]. OSA patients have shown increased platelet activity [104–106] directly proportional to the severity of sleep apnea [107]. Fibrinogen, a major mediator for coagulation, is an acute-phase protein that increases in the body during inflammation [108]. Despite some inconsistent results, most studies indicate that OSA patients have increased fibrinogen levels [54]. Observational studies have shown that the circulating level of fibrinogen is linked to the severity of OSA, as evaluated by the AHI [109, 110]. Thrombin-antithrombin (TAT) complex levels are increased in hypoxic conditions [111] as well as in OSA patients [111, 112]. The level of this complex is used to evaluate fibrin and coagulation levels in the body [113].

The relationship between increased coagulability and OSA in patients may be complicated by many disturbing factors that should be noted [114]. According to a study, the increased risk of VTE in OSA patients is not independent of their body mass index (BMI) [115]. A meta-analysis revealed that patients with obesity have a 2.33-fold increase in VTE occurrence [116]. Weight gain through increased inflammatory activity in the body leads to an increased coagulation factor, fibrinogen, and tissue factor, which provide the basis for developing VTE [117]. Adipocytes have been shown to secrete leptin and IL-6, inflammatory factors leading to increased coagulation activity in obese individuals. Additionally, PAI-1 expression is increased by leptin, leading to an increased risk of thrombosis [118–120].

The results of studies regarding the role of continuous positive airway pressure (CPAP) therapy in reducing inflammatory markers in OSA patients have been conflicting. Although in observational and nonrandomized interventional studies, the use of CPAP led to a decrease in serum inflammatory markers in patients [57, 121]. In a randomized interventional study, treatment with CPAP for four weeks in 100 men with moderate and severe OSA had no significant effect in the decrease of serum levels of inflammatory markers (IL-6, INFγ, and CRP) and adiponectin [122].

Xie et al. [123] performed a meta-analysis to evaluate the effectiveness of CPAP treatment in reducing inflammatory markers (IL-6, 8, CRP, and TNFα) among OSA patients. The effectiveness of CPAP treatment in reducing systemic inflammation in OSA patients has been concluded. The most significant impact was from longer-term use and increased compliance with treatment.

Recently, Friščić et al. [124] investigated the effect of CPAP in reducing the levels of hemogram inflammatory markers in severe OSA patients. In this study, 37 patients who used CPAP properly (for at least 4 h during night sleep) were included, and their inflammatory marker values were assessed before and after at least six months of treatment. In OSA patients, CPAP treatment led to a significant decrease in inflammatory markers, such as neutrophil-to-lymphocyte ratio (NLR) and fibrinogen-albumin ratio (FAR), as revealed by the study's findings. After treatment, patients experienced a reduction in the platelet-to-lymphocyte ratio (PLR) levels; however, there was no significant difference compared to pretreatment values.

### 3.2. Reduced Blood Flow and Venous Stasis in OSA Patients

An increase in blood viscosity and hematocrit in OSA patients has been shown in various studies [117, 125–130]. Blood viscosity is defined as the inherent resistance of blood to flow inside the vessels. Three factors determine blood viscosity: plasma viscosity, hematocrit, and mechanical properties of erythrocytes [128].

Elevated hematocrit and viscosity levels can lead to blood flow disorders and increase the risk of venous stasis in patients [131]. The hypoxia induced in OSA patients is believed to be the main factor at play in this case. Because of hypoxia, the secretion of erythropoietin increases, producing more red blood cells (RBC) [132]. RBC accumulation, which causes an increase in viscosity, blood stasis, and a decrease in nitric oxide (NO) release, leads to enhanced platelet-endothelial activity and, ultimately, thrombosis [133, 134].

Elevated fibrinogen levels in patients with OSA could be linked to increased viscosity. The relationship between increased fibrinogen levels and blood viscosity has been shown in obese people [135]. Many OSA patients are obese, and this should be taken into consideration. Obesity predisposes patients to DVT through more remarkable venous stasis [135, 136].

Intermittent hypoxia causes OSA patients to develop heightened sympathetic activity [137], impaired vascular tonicity, and increased venous stasis, all contributing to more thrombosis [138].

According to a study, OSA patients showed improved rheological variables after undergoing five nights of CPAP treatment compared to the control group. These variables were compared in 31 OSA patients and 19 individuals in the control group before and after CPAP treatment. The results showed that patients with OSA who underwent short-term CPAP treatment significantly improved whole blood viscosity, plasma viscosity, corrected blood viscosity, and the aggregation index [139].

A recent study by Bent et al. [140] revealed that acute CPAP treatment in 16 OSA patients enhanced sleep quality parameters and significantly reduced the patients' whole blood viscosity. The study observed an improvement in RBC aggregation and an increase in plasma viscosity, but the deformability of RBCs did not change significantly. According to their findings, a single CPAP treatment can enhance rheological indices in patients. Other studies also showed a significant effect on improving blood viscosity parameters in OSA patients treated with CPAP [141].

In a study involving animal and human phases, Waltz et al. [142] found results contradicting previous studies. The study showed that rats exposed to severe intermittent hypoxia had an increase in blood viscosity. It is worth mentioning that the rise in hematocrit handles the viscosity surge, with no observed rheological changes in erythrocytes. The human phase of the study revealed that moderate-to-severe OSA patients did not experience obvious rheological disorders. CPAP did not positively affect improving rheological indices in the patients. The authors suggest that the variation in the outcomes of this research and prior studies may be attributed to the patients' selection for the study. In contrast to previous studies that included patients with cardiovascular disease, this study only included OSA patients with normal blood pressure and no evident underlying disease. They concluded that the concurrent presence of comorbidities, rather than OSA alone, may be responsible for the rheological changes observed in OSA patients. Further studies are required to comprehend the inconsistencies in this area with precision.

### 3.3. Endothelial Dysfunction

The exact mechanism is unclear for OSA patients, but hypoxia appears to be a significant factor in this scenario. Chronic intermittent hypoxia-reoxygenation leads to oxidative stress in these patients [143]. Oxidative stress results from an imbalance between oxidant production and antioxidant defense systems [80]. Studies conducted on animals and humans have shown that intermittent hypoxia results in the oxidation of macromolecules and disruption of antioxidant activity [144, 145]. In a meta-analysis conducted on 52 articles, the relationship between OSA and oxidative stress markers was evaluated. The study revealed an imbalanced state of oxidation and antioxidation in OSA patients [146]. Oxidative stress causes the generation of free radicals, including reactive oxygen species (ROS) and reactive nitrogen species (RNS), which results in decreased NO levels and vascular endothelial dysfunction [18, 138, 147, 148]. The reaction between superoxide, a ROS radical, and NO produces proxy nitrite, an RNS. In this reaction, NO is consumed, and its level decreases [149]. NO plays a key role in protecting blood vessels and regulating their tone. Its reduction in blood vessels is associated with vascular dysfunction since it has a significant role as an anti-inflammatory substance [134, 150, 151]. ROS interact with organic molecules, altering their normal functions, changing cellular metabolism, and leading to cellular damage and death [152]. Malondialdehyde (MDA) is a highly reactive end product of lipid peroxidation caused by the attack of ROS on polyunsaturated fatty acids in cell membranes. An elevated serum level of MDA serves as a biomarker for increased oxidative stress in conditions such as OSA [153]. In a systematic review and meta-analysis, Fadaei et al. demonstrated a significant increase in MDA levels in patients with OSA compared to controls. The study also found a positive correlation between MDA levels and the severity of OSA [154]. Mohamed et al. conducted a case-control study on 35 OSA patients and 15 matched healthy subjects to measure oxidative stress biomarkers. The results revealed that patients with OSA express significantly elevated stress biomarkers compared to healthy individuals. Furthermore, a good correlation between all stress markers and polysomnography severity indices was reported [155].

Patients with vascular dysfunction exhibit increased platelet activity and coagulability [50, 156]. Research indicates that patients with OSA have decreased endothelial NO synthase (NOS) expression and increased oxidative stress markers in venous endothelial cells [157]. In various studies, the increase of oxidative stress markers has been shown in OSA patients. In patients with sleep apnea, neutrophil superoxide production increases compared to healthy people [158]. Various human and animal studies have found that intermittent hypoxia leads to the oxidation of macromolecules and the reduction of antioxidant activity [144, 145, 159].

Furthermore, these patients have been found to have increased expression of nicotinamide adenine dinucleotide phosphate (NADPH) oxidase [160], increased levels of lipid [161] and protein [162] peroxidation products, and circulating free DNA [163].

Patients with OSA experience increased levels of procoagulant factors, specifically the soluble cluster of differentiation-40 (CD-40) ligand and soluble P-selectin, which can be attributed to oxidative stress. Platelets are the origin of nearly 95% of soluble CD-40 L [164], which is used to measure platelet activity [165]. P-selectin, mainly expressed by platelets, is key in their activation [166].

Some studies illustrated the link between oxidative stress and endothelial dysfunction by showing the increase in serum levels of markers including Myeloperoxidase (MPO), ICAM-1, VCAM-1, L-selectin, and P-selectin in OSA patients [167, 168]. VE-cadherin cleavage is another mechanism of vascular impairment, and the increase in the circulating serum level of VE-cadherin indicates endothelial dysfunction and heightened vascular permeability. The process involved is related to oxidative stress in OSA patients [169].

The fibrinolytic system in the human body stops the growth of clots and obstructions inside blood vessels. The transformation of plasminogen into plasmin leads to the breakdown of fibrin and the dissolution of blood clots. PAI-1 is produced by various cells. Approximately 10 percent was present in the bloodstream and the subendothelial matrix; the rest was stored in platelets [170]. Different studies have shown that the level of PAI-1 increased in oxidative stress [171], and the administration of antioxidants decreased its levels [172, 173]. HIF-1α is not active under normal oxygen conditions but triggers PAI-1 transcription when activated under hypoxic conditions. Disturbance in tissue oxygenation and the resulting oxidative stress lead to the production of ROS from endothelial cells, the increase of HIF-1α activity, and the increase of PAI-1 [174]. PAI-1 inhibits NOS, leading to a decrease in NO levels. As a result, PAI-1 acts as a mediator of endothelial dysfunction [27].

In OSA, a complex network of links between the oxidation-antioxidation system and inflammatory pathways was described [175]. The relationship between oxidative stress and increased inflammatory activity, as well as increased activity of HIF-1α and NF-κB pathways, has been demonstrated by Ryan et al. [176]. Elevated serum levels of ROS, besides NO depletion, lead to endothelial cell damage and, ultimately, activation of various cellular signaling pathways, such as IκB kinase. Phosphorylation of the IκB and its degradation leads to the release of NF-κB and translocation to the cell nucleus, where the transcript of the multiple genes involved in the inflammatory pathways. Consequently, proinflammatory cytokines (TNF-α, IL-6, and IL-1β) generate and release [177–179]. As mentioned earlier, elevated IL-6 and TNF-α increase PAI-1 in the body [103, 180, 181]. On the other hand, the NF-κB pathway with the production of ROS enhances oxidative stress and is partly responsible for establishing a vicious cycle of cellular impairment [182].

Various studies have shown that CPAP therapy improves endothelial function [183–185]. Xu et al. conducted a meta-analysis using the random effect model. The study found that CPAP uses significantly improved endothelial function when evaluated by flow-mediated dilation but not by the nitroglycerin-mediated dilation (NMD) method [186].

In a study, Kohler and his colleagues showed that OSA patients who stopped using CPAP for 2 weeks had a decrease in endothelial function compared to the group that continued using CPAP. This study assessed the endothelial function using flow-mediated dilation [187].

**Figure 2:**
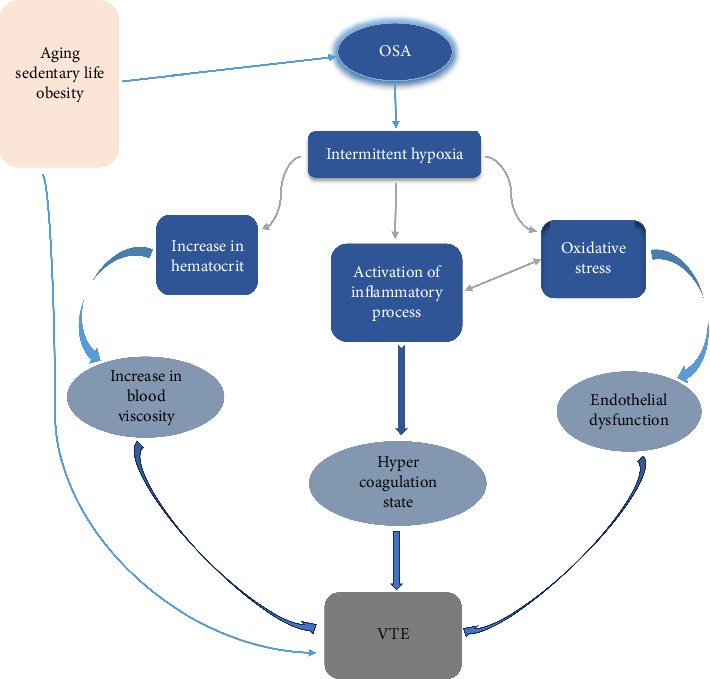
Summary of venous thromboembolism (VTE) pathogenesis in obstructive sleep apnea (OSA).

Based on the information mentioned above, in the pathogenesis of VTE among patients with OSA, intermittent chronic hypoxia plays a central role. In addition to the common risk factors for vascular thrombosis—such as age, obesity, and reduced physical activity—these factors contribute significantly to the development of VTE in OSA patients. Repetitive upper airway obstruction causes oxygenation-reoxygenation events that promote oxidative stress and the generation of free radicals. The free radicals can directly damage vascular integrity and cause endothelial dysfunction. Furthermore, activating inflammatory pathways in hypoxemic conditions leads to the secretion of various proinflammatory and procoagulant factors. The interrelation between oxidative stress and the inflammatory process was noted. Additionally, a hyperviscosity state and venous stasis were established due to hypoxemia (Figure 2).

## 4. VTE Recurrence in OSA Patients

Patients who experience the first episode of VTE are at risk of recurrence. The likelihood is higher in the first year after the initial incident [188]. The cumulative risk of VTE recurrence in the first 5 years is 25% [165]. According to reports, patients with a recurrence of pulmonary emboli may experience mortality rates as high as 9% [189]. Anticoagulant treatment should be administered to prevent the spread and recurrence of primary thrombosis [172, 190]. It is recommended that VTE be treated for 3 months, and the decision to continue treatment after this time depends on the rate of disease recurrence [191]. Various factors impact the recurrence of VTE in patients. To estimate the risk of VTE recurrence, different demographic, clinical, and laboratory characteristics should be considered [190]. In various studies, active cancer, male gender, paresis of the lower limbs, increasing age, increased BMI, hereditary thrombophilia, antiphospholipid syndrome, and continuous increase in D-Dimer levels are associated with VTE recurrence [166, 190, 192]. Limited information is available on the frequency of VTE recurrence in OSA patients. The findings of longitudinal observational studies have been contradictory (Table 1).

For 5–8 years, Alonso-Fernández et al. [193] monitored 120 patients who stopped taking oral anticoagulants after their first VTE episode. VTE recurrence was experienced by 19 patients in the study, out of which 16 had AHI ≥ 10. The research showed that AHI ≥ 10 had an independent effect on the recurrence of PE. Besides the AHI, nocturnal hypoxia was also an independent risk factor for PE recurrence. Despite adjusting for confounding factors such as the BMI, this study still found OSA to be an independent risk factor for VTE recurrence. On the other hand, the high incidence of obesity among OSA patients may also play a part in VTE recurrence in these patients. There could be a synergistic effect between obesity and OSA in increasing the probability of VTE recurrence in these patients. It was also shown that nocturnal hypoxemia was associated with an increase in the likelihood of VTE recurrence in patients. As mentioned earlier, the pathogenesis of VTE in OSA patients is primarily caused by intermittent hypoxia, and it may also impact thrombotic event recurrence.

Xie et al. [194] found some consistency with the previous study. Of the 97 patients with PE included in the study, 32 had OSA and were monitored for 18 months. Patients with OSA had a significantly higher recurrence rate of PE, with a 21% occurrence compared to 6% in those without sleep apnea.

Seckin et al. [17] evaluated the hospitalization information of 25,038 patients in a retrospective study. Among these, 283 patients had PE during admission or hospitalization. The rate of OSA in PE patients was 2.4% compared to 0.9% in the group without OSA (OR, 2.51: 95% CI 1.9–3.3). Despite the increased risk of acute PE in OSA patients, the risk of death did not increase. In the 10-year follow-up of the patients, it was shown that the only known risk factor in the patients was OSA. Although the recurrence of PE in patients treated with CPAP decreased by 30%, a significant difference has not been detected, possibly due to the study's small sample size.

In a cohort study conducted in France, Nepveu and colleagues followed up on 2109 patients with acute VTE after discontinuation of anticoagulant agents. Of these, 74 patients had OSA. During the follow-up, 252 patients experienced VTE recurrence, among which nine had OSA. The study revealed no significant correlation between OSA, CPAP treatment, O2 saturation, and AHI with VTE recurrence. The risk of VTE recurrence was higher in patients who developed OSA during follow-up after primary VTE compared to those with a history of OSA prior to VTE diagnosis [196].

In another study on patients with PE from RIETE registry data, 241 of 4153 patients had a history of OSA. The statistical analysis indicated that pre-existing OSA at 30, 90 days, and 1-year follow-up was not significantly associated with the recurrence of thromboembolism or the occurrence of major bleeding. Patients with an OSA history had a higher mortality risk because of PE as compared to those without OSA [195].

## 5. Discussion

OSA has been identified as a potential independent risk factor for VTE, and the risk increased directly with OSA severity. Raj et al., in a systematic review, revealed a statistically significant link between OSA and VTE. Also, it was identified that patients with severe OSA who needed CPAP therapy were at a higher risk for developmental consequent VTE [131]. We reviewed available studies explaining the pathogenesis of this relationship in OSA patients. This pathogenesis's foundation stone is according to the development of intermittent hypoxia in this condition. The interrelation between inflammation and oxidative stress and enhanced blood viscosity contribute to the three sides of Virchow's triad and increase patients' vulnerability to VTE. Unfortunately, despite relatively adequate studies on the relationship between OSA and VTE, our findings about VTE recurrence in OSA patients are limited. Currently, the results of studies conducted to obtain a precise answer to our clinical question regarding the increased likelihood of VTE recurrence in OSA patients have been quite contradictory. Variations in study design, such as whether they are prospective or retrospective, differences in patient populations, and levels of CPAP adherence, may explain these discrepancies. As previously mentioned and illustrated in the relevant table, the varying number of study populations and the differing durations of patient follow-up are noteworthy when reviewing the studies conducted in this field. Most studies focused solely on patients with PE, but one study also included patients with primary DVT. It is important to note that the initial treatment duration for VTE varied across studies, ranging from less than 3 months to over 6 months. This variation in treatment duration may influence the recurrence rate of VTE after discontinuing anticoagulants.

However, in 2023, a meta-analysis was published that examined the effects of OSA on the clinical outcomes of patients with PE. Based on the results, the incidence of recurrent PE was significantly higher in OSA patients than in controls (OR: 2.68; 95% CI 1.61, 4.97 *I*^2^ = 0%, *p* < 0.00001). Although this result was evaluated from only three studies, it should be considered in the long-term clinical management of patients with OSA and PE [197].

Another important issue that needs to be addressed is the role of CPAP therapy in preventing VTE recurrence in OSA patients. It is assumed that CPAP therapy reduces thrombosis by partially improving intermittent hypoxia and regressing the pathogenesis of VTE [131]. The first part of this hypothesis was evaluated in the umbrella review of meta-analyses by Fiedorczuk et al. The study was conducted regarding the potential utility and monitoring of serum biomarkers in patients with OSA. They concluded that OSA is associated with elevated levels of inflammatory cytokines, oxidative stress indicators, and adhesion molecules, which can be decreased using CPAP [198]. Is there any evidence about the second part of the above hypothesis? Does reducing serum inflammatory biomarkers in patients using CPAP lead to diminished VTE incidence at the patient's bedside? The lack of sufficient studies in this field is quite striking.

In the study by Seckin et al. [17], a reduction in VTE recurrence was observed in patients receiving CPAP, although this result was not statistically significant. Although using CPAP based on the aforementioned hypothesis would likely reduce the incidence of thrombosis in patients, there is currently insufficient evidence to support this.

Although they have long been viewed as distinct conditions, new evidence suggests that VTE and atherothrombosis may be linked by a shared pathophysiological mechanism in which inflammation plays a key role. Piazza and Ridker proposed VTE as a chronic inflammatory disorder [199]. Currently, it has even been suggested that VTE should be considered as part of the pan-cardiovascular syndrome [200]. In a meta-analysis, it has been shown that the risk factors of cardiovascular diseases such as obesity, HTN, and diabetes mellitus (DM) have been associated with an increased risk of VTE in patients [116]. It has also been shown in various studies that in the follow-up of patients with VTE, they are at an increased risk of cardiovascular diseases [14, 201, 202]. As mentioned earlier, OSA patients have a chronic inflammatory condition in the body, which increases the risk of cardiovascular diseases and VTE. Antiplatelet and anti-inflammatory treatments are used in the treatment of cardiovascular diseases due to the role of inflammation in the pathogenesis of this disorder. The primary question is whether the chronic inflammatory process in OSA necessitates long-term treatment for VTE, similar to cardiovascular diseases. Should we approach this disease as an acute time-dependent disorder with short-term antithrombosis treatment or a chronic disorder that needs long-term anti-inflammatory treatment?

We currently face a scientific gap in answering these clinical questions. To solve these dilemmas and appropriately address the problems in OSA patients, we need to design and implement more studies in the future. Future studies should be prospectively designed with larger sample sizes and extended follow-up periods. The role of CPAP in the rate of VTE recurrence should also be considered and evaluated.

The primary limitation of previous studies in this field is the small sample size, along with the limited number of articles. Another noteworthy point is the heterogeneity of studies regarding the patient's follow-up length. It is important to note that the study with the most significant participants had the lowest clinical follow-up regarding recurrence. Given that the development of intravascular thrombosis depends on the progression of inflammation and is a gradual and chronic process, it is not unreasonable to assume that there will be no recurrence of VTE in a relatively short follow-up.

## 6. Conclusion

Besides traditional risk factors, intermittent hypoxia caused by frequent obstruction of the upper airways during sleep leads to activation of the inflammatory system, oxidative stress, and increased viscosity in OSA patients. The development of the mentioned cases in OSA patients provides the basis for developing VTE and its recurrence. The results of previous studies on VTE recurrence in OSA patients are conflicting. Although the use of CPAP leads to diminished proinflammatory cytokines and oxidative stress indicators, there is currently insufficient clinical evidence to support that this therapy can prevent recurrent VTE in patients with OSA. We currently face a scientific and practical gap regarding the potential risk of recurrent VTE in OSA patients and the role of CPAP in managing patients to reduce this risk. Given that the duration of treatment for VTE is determined by the rate of its recurrence in patients, we need to resolve the abovementioned complex issue to make decisions for better patient management. The authors strongly recommend the design and implementation of prospective studies with larger sample sizes and longer clinical follow-up periods, as well as paying special attention to the role of CPAP treatment in these studies in the future.

## Figures and Tables

**Table 1:** Description of the longitudinal studies on VTE recurrence in OSA patients.

Author	Overall participants	Participants with OSA	Duration of follow-up	Findings on VTE recurrence	Other findings
Xie et al. [194]	97 patients with PE, with at least 6 months of treatment with warfarin	32 patients had OSA	12 months, prospective	OSA patients had more recurrence of PE (21.43% vs. 6.78%, *p*=0.047).	No significant difference was seen in terms of the total number of adverse effects after warfarin termination (28.57% vs. 16.95%, *p*=0.214).
Alonso-Fernández et al. [193]	120 patients with a first episode of PE who had received at least 3 months of OAC	71 patients with PE (59.2%) had OSA	78 ± 16 months, prospective	Among 19 patients presented with a PE recurrence, 16 had OSA. Crude hazard ratio (HR), 4.05; 95% CI, 1.18–13.91; *p* 0.026	9 patients died, none because of thromboembolic events. OSA was not related to more frequent mortality.
Seckin et al. [17]	283 patients with PE	75 patients with OSA	4 and a half years, retrospective	Patients with OSA had a higher risk of PE recurrence (OR 2.16, 95% CI 1.17–3.99, *p*=0.015).	OSA was not a significant determining factor for mortality who experienced a PE (OR, 0.56; 95% CI, 0.11–2.78; *p*=0.47)
Le Mao et al. [195]	4153 patients with PE	241 patients with preexistence OSA	1 year follow-up, prospective	OSA was not significantly associated with 30-day recurrent venous thromboembolism (adjusted OR: 0.6; 95% CI: 0.1–4.7; *p*=0.65) or major bleeds (adjusted OR: 1.0; 95% CI: 0.4–2.2; *p*=1.0). Similar findings were reported at 90 day and 1-year follow-ups.	OSA was not a significant predictor of all-cause mortality (odds ratio (OR): 1.5; 95% CI: 0.8–2.9; *p*=0.19). However, patients with pre-existing OSA related to increased PE-specific mortality (adjusted OR: 3.0; 95% CI: 1.3–6.8; *p*=0.01).
Nepveu et al. [196]	2109 patients with documented VTE, with at least 3–6 months of OAC	74 patients had moderate-to-severe OSA	4.8 years, prospective	The recurrence risk was not increased in patients with OSA	The risk of mortality was not increased in OSA patients

Abbreviations: OAC = oral anticoagulation, OSA = obstructive sleep apnea, PE = pulmonary embolism, and VTE = venous thromboembolism.

## Data Availability

The authors confirm that the data supporting the findings of this study are available within the article.
